# Analysis and forecasting of syphilis trends in mainland China based on hybrid time series models

**DOI:** 10.1017/S0950268824000694

**Published:** 2024-05-27

**Authors:** Zhen D. Wang, Chun X. Yang, Sheng K. Zhang, Yong B. Wang, Zhen Xu, Zi J. Feng

**Affiliations:** 1 School of Public Health, Shandong Second University, Weifang, China; 2 School of Basic Medicine, Institute of Basic Medical Sciences, Chinese Academy of Medical Sciences, Peking Union Medical College, Beijing, China; 3 School of Public Health, Xinxiang Medical University, Xinxiang, China; 4 National Key Laboratory of Intelligent Tracking and Forecasting For Infectious Diseases, Chinese Center for Disease Control and Prevention, Beijing, China; 5 Chinese Preventive Medicine Association, Beijing, China

**Keywords:** LSTM, modelling, NARX, SARIMA, syphilis

## Abstract

Syphilis remains a serious public health problem in mainland China that requires attention, modelling to describe and predict its prevalence patterns can help the government to develop more scientific interventions. The seasonal autoregressive integrated moving average (SARIMA) model, long short-term memory network (LSTM) model, hybrid SARIMA-LSTM model, and hybrid SARIMA-nonlinear auto-regressive models with exogenous inputs (SARIMA-NARX) model were used to simulate the time series data of the syphilis incidence from January 2004 to November 2023 respectively. Compared to the SARIMA, LSTM, and SARIMA-LSTM models, the median absolute deviation (MAD) value of the SARIMA-NARX model decreases by 352.69%, 4.98%, and 3.73%, respectively. The mean absolute percentage error (MAPE) value decreases by 73.7%, 23.46%, and 13.06%, respectively. The root mean square error (RMSE) value decreases by 68.02%, 26.68%, and 23.78%, respectively. The mean absolute error (MAE) value decreases by 70.90%, 23.00%, and 21.80%, respectively. The hybrid SARIMA-NARX and SARIMA-LSTM methods predict syphilis cases more accurately than the basic SARIMA and LSTM methods, so that can be used for governments to develop long-term syphilis prevention and control programs. In addition, the predicted cases still maintain a fairly high level of incidence, so there is an urgent need to develop more comprehensive prevention strategies.

## Introduction

Syphilis, an infectious disease caused by the bacterium *Treponema pallidum*, is a preventable and treatable condition predominantly transmitted through sexual contact, including oral, vaginal, and anal intercourse. Additionally, vertical transmission can occur from mother to foetus during pregnancy, and less commonly, the disease may be spread through blood transfusion. The clinical presentation of syphilis can be asymptomatic [1], rendering the identification of infected individuals challenging in the absence of serological screening. Consequently, routine testing is imperative for the detection of syphilis, particularly in populations at increased risk for sexually transmitted diseases (STDs) [2, 3]. Syphilis can result in the development of genital ulcers, pain, and inflammation. Untreated, the disease can advance to affect various organs and systems. Advanced syphilis can lead to detrimental effects on the heart, major blood vessels, central nervous system, and skeletal structure, resulting in a myriad of complications such as heart valve abnormalities, meningitis, stroke, optic nerve damage, and bone degeneration.

In recent decades, the number of syphilis cases has been increasing [4]. An estimated 5.7–6 million new cases are detected annually worldwide among individuals aged 15–49 years [5]. Between 2016 and 2023, the annual reported incidence rate of congenital syphilis ranged from 700 000 to 1.5 million cases per year [6] and the case-fatality rate (CFR) among offspring of pregnant women with syphilis was 31% [7]. The reported incidence of syphilis in China escalated from 4.50 per 100 000 in 2003 to 34.04 per 100 000 in 2021 [8] and the mortality rate from syphilis was recorded at 0.002 per 100 000 individuals [9]. Syphilis has become the highest number of reported incidences of all STDs in mainland China [10]. As a serious public health problem, it has attracted great attention from the national health authorities. A prerequisite for policymakers to develop policies is to make scientific forecasts of disease trends. Many approaches are available for modelling and forecasting time series (TS) data. The most widely used TS model is the autoregressive integrated moving average (ARIMA) model [11, 12], a prerequisite for the applicability of ARIMA models is the requirement that the TS data should be stable, so ARIMA models tend to be poor fits for nonlinear data, the real TS data tend to be more complex, containing both nonlinear and linear components. For the nonlinear component of the model, Machine Learning (ML) is a more applicable approach. ML is a field that focuses on the learning aspect of Artificial Intelligence (AI) by developing algorithms that best represent a set of data [13]. Originally inspired by neurobiology, deep neural network models have become a powerful tool of ML and artificial intelligence. They can approximate functions and dynamics by learning from examples [14]. Artificial Neural Networks (ANN) are an important part of ML, which is an autonomous computational project designed by imitating the structure of biological neurons. According to the different topologies of neural networks, artificial neural networks can be divided into feed-forward, feedback, and recurrent neural networks (RNNs), among which the RNN models, such as the long short-term memory network (LSTM) models, the nonlinear auto-regressive models with exogenous inputs (NARX) have unique advantages in processing TS data, this is because the structure of the RNN models determines that the output at moment *t* is not only related to the input at moment *t* but related to the output at moment *t −* 1. LSTM, leveraging its gating mechanism and memory unit, is capable of capturing prolonged dependencies within sequential data and conducting contextual modelling, thereby exhibiting remarkable efficacy in the modelling and prediction of sequential data [15]. The NARX neural network is a dynamic neural network that incorporates delay and feedback mechanisms, thereby enhancing its ability to memorize historical data. It is suitable for simulating and predicting nonlinear time-series data in multiple domains [16]. Compared to other data-driven models, the NARX model demonstrates strong problem-solving capabilities, fast convergence speed, and high prediction accuracy when handling seasonal TS forecasting [17]. Since ARIMA models have a good ability to fit the linear component of TS data, many researchers combine the ARIMA models and RNN models into hybrid ARIMA-RNN models to predict the convergence of the TS data, and the prediction results of the hybrid models are often better than those of single ARIMA or RNN models [18–23]. In this study, we simulated the TS data of syphilis incidence using a single ARIMA model, LSTM model, hybrid ARIMA-LSTM model, and hybrid ARIMA-NARX model, respectively, both models were used to make predictions with a period of 12. The fit and prediction indicators were calculated separately to evaluate the performance of these models.

## Methods

### Data collection

The monthly number of newly reported cases of syphilis from January 2004 to November 2023 was obtained from the website of the Bureau for Disease Control and Prevention of China National Health Commission (http://www.nhc.gov.cn/jkj/new_index.shtml) by searching for the Chinese translation of the keyword ‘*Overview of National Notifiable Infectious Diseases Epidemic*’ in the website’s search box. The monthly reports were simultaneously published on the China National Knowledge Infrastructure (CNKI, https://www.cnki.net/). The case information of notifiable infectious diseases was timely reported from local hospitals and community health service centres throughout the country and was reviewed and confirmed by local Centers for Disease Control and Prevention (*CDC*) after confirmatory tests [24]. A total of *N* = 239 observations were included in the study (Table S1).

### TS decomposition

TS decomposition is a technique to break down a TS into its underlying components. Which is expressed as *Yt = T_t_ + S_t_ + I_t_*, where *T_t_*, *S_t_*, and *I_t_* denote the trend, seasonal component, and a stochastic irregular component, respectively. We performed the Mann–Kendall (M–K) test for the trend. As the sample data is monthly, we confirmed the *T_t_* by using a smooth weighted 13-term moving average filter given by:

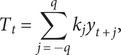


*q = 6* for monthly data*, q < t < N − q, k_j_ = 1/4q* for *j = ± q*, and *k_j_ = 1/2q* otherwise. After the transformation of TS, the first and last *q* observations were lost, so we repeated the first and last smoothed values *q* times.

Let *n_t_* be the total number of observations made in period *t*, the stable seasonal filter is given by

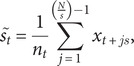




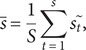




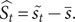

For *s* = 12, *t* = 1,…, *s.* Using 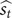 to constrain the seasonality component to fluctuate around *zero.*

### Modelling of SARIMA

#### Mathematical equations of the SARIMA model

The SARIMA model is always expressed as SARIMA *(p, d, q) (P, D, Q)_s_*, where *p, d, and q* represent non-seasonal components, and *P, D, and Q* represent seasonal components. *p* and *P* are lags of non-seasonal and seasonal autoregressive, respectively. *d and D* are degrees of non-seasonal and seasonal differencing, respectively. *q* and *Q* are lags of the non-seasonal and seasonal moving averages, respectively, and *s* denotes the periodicity.

The polynomial of SARIMA *(p, d, q) (P, D, Q)_s_* model can be expressed as

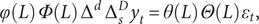




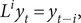




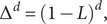




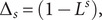




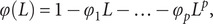




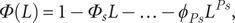




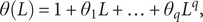




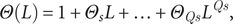

where 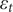 denotes a sequence of uncorrelated random variables from a defined probability distribution with a mean zero.

#### Constructing the SARIMA model

Initially, we conducted an Augmented Dickey–Fuller (ADF) test to determine the lags of seasonal and non-seasonal differencing required to achieve data stationarity. Subsequently, we established a range for the *p*, *q*, *P*, *Q* parameters, from 0 to 4. We computed the Akaike Information Criterion (AIC) and the Bayesian Information Criterion (BIC) for each permutation of the SARIMA model. The model exhibiting the lowest combined sum of AIC and BIC was selected as the optimal SARIMA model and the parameters of the model were estimated by the maximum likelihood approach. We conducted a Ljung–Box Q-test, along with the ACF and PACF plots on the residuals to check the autocorrelation. Besides, we performed normality diagnostics by plotting the histogram of standard residuals and the Quantile-Quantile (QQ) plot of residuals. The TS was divided into a training set (the first 227 observations) for modelling and a test set (the last 12 observations) for predicting. Finally, the simulation performance of the training set and the test set were calculated separately.

### Constructing a single LSTM model

#### Structure and equation of LSTM neural network

**Figure 1.**
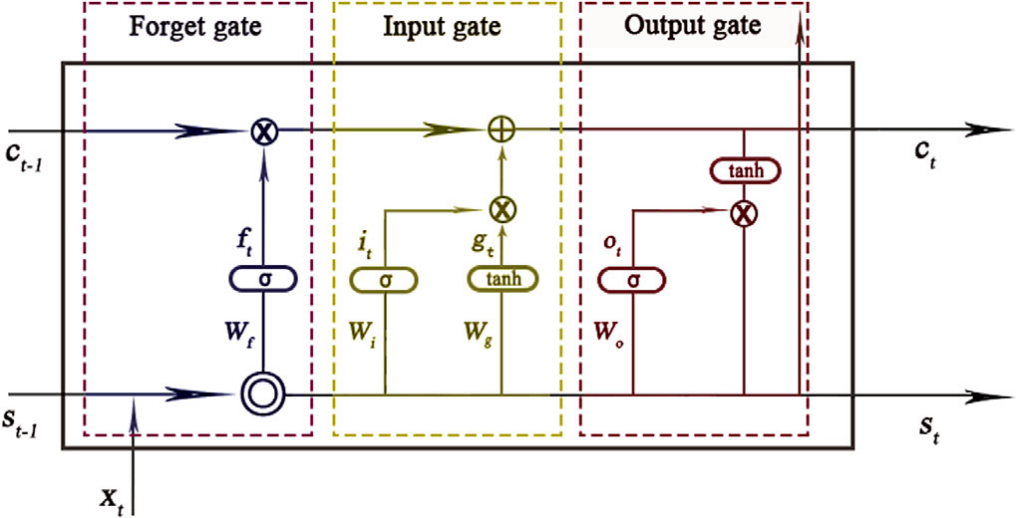
The cell structure of the LSTM network. *Note:* The arrow indicates the data flow, where *x*, *s*, *c*, *f*, *i*, *g*, and *o* denote the input, output, cell state, forget gate, input gate, cell candidate, and output gate in time step *t*, respectively. *σ* and *tanh* denote the *sigmoid* activation function and the hyperbolic tangent function, which maps the data to (0,1) and (−1,1), respectively. 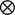 are vector operators which represent element-wise multiplication and element-wise addition, respectively

The LSTM network is a kind of RNN consisting of a sequence input layer, an LSTM layer, and an output layer. The sequence input layer inputs TS data into the network, and the LSTM layer learns long-term dependencies between time steps of sequence data. Different from the traditional RNN, there is a cell state in the LSTM layer, which can effectively keep the long-term information learned from the previous time steps and solve the problem of gradient disappearance. At each time step, the layer adds information to or removes information from the cell state, all these updates are controlled by gates. There are three kinds of gates in the LSTM layer, input gate (*i*), forget gate (*f*), and output gate (*o*). Figure 1 illustrates the flow of data at time step *t* and shows how the gates forget, update, and output the cell and hidden states.

The following formulas describe the operation of the data in the LSTM layers at time step *t.*


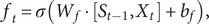




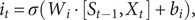




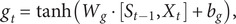




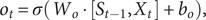




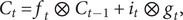




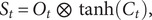

where *W* and *b* denote the matrices of input weight and bias, respectively.

#### Training and simulation of the LSTM model

It is necessary to standardize the data before modelling to eliminate the effects of abnormal values and improve the speed of model convergence. A *z*-score method was used to normalize the TS data, which was given by TS* = (TS − μ)/σ, where *μ* and *σ* denote the mean and standard deviation of the TS data. To prevent the gradients from exploding, we set the gradient threshold of the network to 1. The loss function is an important basis for adjusting parameters, it reflects the difference between the original and predicted values during the training, if the loss function decreases too slowly at the initial stage of training, it may mean that the number of hidden neurons or the value of the learning rate is set too small. We used the *Adam* solver to update the network parameters by taking small steps in the direction of the negative gradient of the loss function. The solvers update the parameters using a subset of the data at each step.

The varying quantities of hidden neurons, iterations, and learning rates can all influence the simulation performance of the model. Therefore, a commonly used method for adjusting parameters involves fixing the learning rate and iteration count, and then conducting model training with different numbers of neurons [16]. Presently, there is no mature theoretical evidence for determining the optimal number of neurons. Consequently, the majority of studies rely on trial and error [25]. In our study, we set the initial learning rate to 0.005, which is the median value of recommended [26], the max iterations was set to 500, and the number of hidden neurons in increments of 10, ranged from 10 to 200. To automatically drop the learning rate during training, using a piecewise learn rate schedule, multiply the initial learning rate by a drop factor of 0.2 after half of the maximum iterations. To mitigate the risk of overfitting, we incorporated L2 regularization and implemented dropout layers within the model. We then calculated the goodness-of-fit for the test set under different numbers of neurons, using the principle of minimizing the root mean square error (RMSE) to determine the best-fitting LSTM model. The first 226 data of the training set were set as input of the model, and the data shifted to one-time step were set as output of the model. A 12-step forward prediction was then performed using the trained LSTM model. Finally, the simulation performance of the training set and the test set were calculated separately.

#### Constructing a hybrid SARIMA-LSTM model

The thought of constructing the hybrid model is to express the link between the output of SARIMA and original observations (i.e., the residuals of the SARIMA model) by an LSTM model, for the residuals of the SARIMA model containing the time information and random fluctuations. Although the SARIMA model effectively captures the linear components of the TS data, its capability to capture non-linear features is comparatively limited when compared to the LSTM model. Consequently, the residuals of the SARIMA model may contain unutilized random fluctuation information from the original data. One of the key advantages of the LSTM model lies in its capacity to model stochastic data. Leveraging this capability, we employed the LSTM approach to re-model the residuals derived from the SARIMA model. Subsequently, we integrated the LSTM model’s output with that of the SARIMA model to obtain the hybrid SARIMA-LSTM model’s output. The modelling and prediction processes remained consistent with those of the single LSTM model. In our study, we set the initial learning rate to 0.005, the max iterations was set to 500, and the number of hidden neurons ranged from 10 to 200 in increments of 10. To automatically drop the learning rate during training, using a piecewise learn rate schedule, multiply the initial learning rate by a drop factor of 0.2 after half of the maximum iterations. To mitigate the risk of overfitting, we incorporated L2 regularization and implemented dropout layers within the model. We then calculated the goodness-of-fit for the test set under different numbers of neurons, using the principle of minimizing the RMSE to determine the best-fitting SARIMA-LSTM model. Subsequently, a 12-step forward prediction was executed using the trained SARIMA-LSTM model, and the simulation performance of the training and test sets was evaluated separately.

#### Constructing a hybrid SARIMA-NARX model

The NARX network is a powerful neural network architecture specifically designed for modelling and predicting TS data by considering both the autoregressive relationship within the TS and the influence of exogenous inputs.

The NARX network consists of two main components: the autoregressive (AR) part and the exogenous (X) part. The AR part captures the relationship between past values of the TS itself, while the X part captures the influence of the exogenous inputs on the TS. The X part can be implemented as a separate input layer or concatenated with the AR inputs.

During training, the NARX network is fed with historical data, including both the TS values and the corresponding exogenous inputs. The network learns to predict the future values of the TS based on its past values and the exogenous inputs. The training process involves adjusting the network’s weights and biases to minimize prediction errors.

The defining equation for the NARX model is

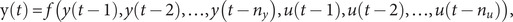

where *f* represents a function that relies on the structure and connection weights of the NARX model, *y* refers to the sample TS data in a lagged period *d*, and *u* refers to the input series containing the time factor and the projections of the SARIMA model, y is the simulation values by the hybrid SARIMA-NARX model at time step *t.* Before modelling, we need to define the structure of the model. In this model, the simulated series of the SARIMA model was treated as the input, while the corresponding reported cases of syphilis were regarded as the output. Subsequently, we randomly divided the data into a training set, a validation set, and a test set in the ratio of 80%, 10%, and 10% respectively [27]. Since the delays of the input and the number of hidden neurons have an impact on the performance of the model, we constructed multiple open-loop (series–parallel) architectures containing different number of hidden neurons (experimented from 2 to 40) for training the networks separately, using the Levenberg–Marquardt algorithm for updating weights during training. We evaluated the goodness-of fit of models under different numbers of neurons and calculated the RMSE for both the training and test sets. The SARIMA-NARX model with the smallest RMSE value on the test set was chosen as the best-fitting model. Finally, the trained open-loop network was transformed into a closed-loop (parallel) architecture to make a 12-step-ahead forecast and the goodness-of-fit for the training and test sets were calculated separately.

### Goodness-of-fit checks of models

The *R* ^2^, median absolute deviation (MAD), RMSE, mean absolute percentage error (MAPE), and mean absolute error (MAE) of train set and test set were used as indicators for evaluating the simulation and prediction performance of the models mentioned above, which were given by

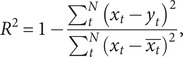




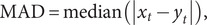




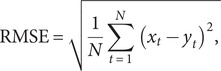




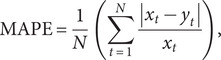




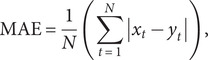

where 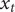 and 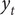 denote the original series and fitting series, respectively.

### Software and significant level

MATLAB 2023a (MathWorks Corporation, Natick, MA) was used to perform the models involved in the study, and Microsoft Office 2021 (Microsoft Corporation, Natick, MA) for data collection and processing. A two-sided *p* < 0.05 was considered statistically significant.

### Ethical review

The study protocol and utilization of syphilis incidence data were obtained from the Bureau for Disease Control and Prevention of China National Health Commission and no ethical issues were identified. Therefore, an ethical statement was not necessary because the data are public access data.

## Results

### Trends and seasonality of the sample data

**Figure 2.**
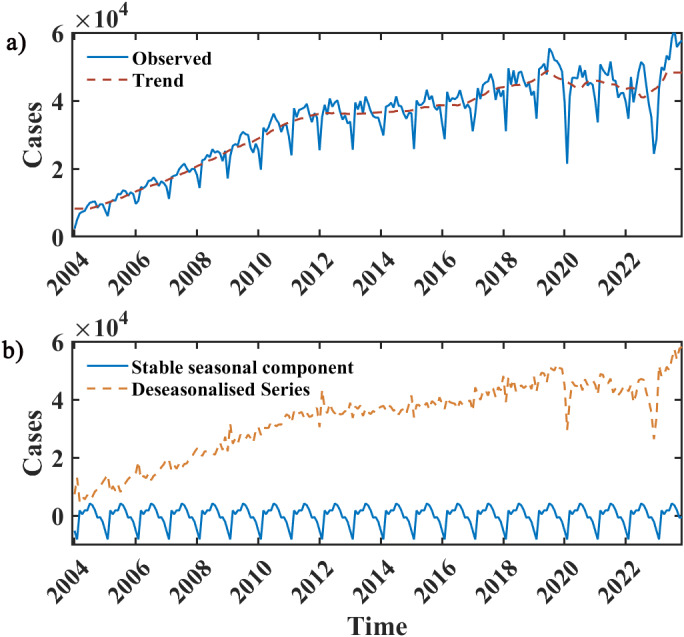
Monthly reported cases of syphilis from January 2004 to November 2023 and the decomposition of the TS data. *Note:* In (a), the blue curve depicts the monthly reported incidences of syphilis, while the red curve illustrates the long-term trend. Meanwhile, in (b), the blue curve represents the stable seasonal component exhibiting a periodicity of 12 months, and the yellow dash curve portrays the time series post-seasonal component extraction

The monthly average number of reported cases for the last 3 years was 45 735, which is 26.74% higher than the average for the whole period. The peak number of reported incidents was 61 068 in November 2023. The M–K test results indicate an overall increasing trend in the data (*z* = 17.11, *p* < 0.05). As shown in Figure 2a, the number of syphilis infections trended upward from 2004 until Quarter 3 of the year 2019, monthly reported cases increased at a higher rate from 2004 to 2012 than from 2013 to 2019 and has levelled off since then, however, no significant downward trend has been observed since the year 2019 (*z* = 0.31, *p* > 0.05). The decomposition results of the data showed a periodicity of 12 in the TS data, with a peek number of incidences in July, and less prevalence in winter and spring than in summer and autumn (Figure 2b).

### The best-fitting SARIMA model

The ADF test results indicate that the TS data achieves stationarity subsequent to the implementation of both a first-order differencing and a first-order seasonal differencing (*t* = −7.22, *p* < 0.01), therefore *d* and *D* of the SARIMA model should be set to 1. In the process of model selection, the SARIMA(4, 1, 0)(4, 1, 0)_12_ emerged as the superior model, as evidenced by the minimization of the combined AIC and BIC values (AIC = 4 072.3, BIC = 4 102.6) relative to competing models. Consequently, this SARIMA model is adjudged to be the optimal fit for the TS data under study. The best-fitting SARIMA model can be expressed as a polynomial of

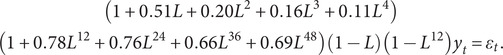



**Figure 3.**
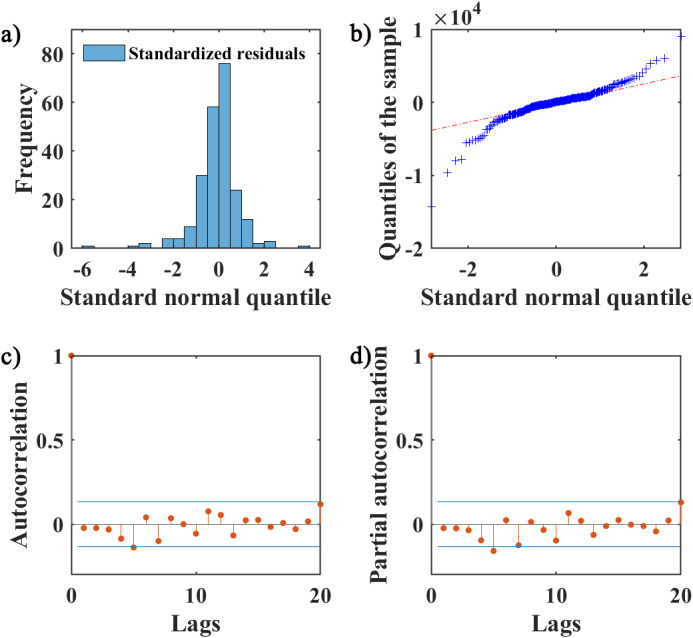
SARIMA model residuals normality and autocorrelation diagnostics. *Note:* (a) The frequency distribution of standardized residuals using a histogram. (b) The QQ plots of residuals of the SARIMA model, the red dashed line represents the standard normal distribution. (c,d) The ACF and PACF of residuals, respectively. The stem plots represent the values of ACF and PACF at different lags, and the blue lines indicate the ±2 times standard deviation interval

We performed the autoregression and normality diagnostics on the residual series, and the result of Ljung–Box Q-Test test showed that there was no autocorrelation in the residuals (*χ* ^2^ = 17.81, *p* = 0.59), and the residual ACF and PACF plots showed that most of the residuals were within the ±2 times standard deviation interval, which indicated that the fitting was successful. The histogram of the standardized residual distribution and the QQ plot of the residuals indicated that the standardized residuals showed an almost symmetrical distribution with zero as the boundary, and the frequency of the standardized residuals in the ±2 interval accounted for more than 80% of all, which can therefore be regarded as a normal distribution (Figure 3).

A 12-time-step prediction was performed using the SARIMA(4, 1, 0)(4, 1, 0)_12_ model, The fitting and predicting efficacy of the model was calculated separately, which are shown in Table 1.

**Table 1.** Evaluation of goodness-of-fit of SARIMA, LSTM, SARIMA-LSTM, and SARIMA-NARX models

Models	Fitting power					Predicting power				
	*R* ^2^	MAD	MAPE	RMSE	MAE	*R* ^2^	MAD	MAPE	RMSE	MAE
SARIMA	96.54%	869.64	5.13%	2 363.59	1 504.26	18.99%	13 643.14	28.72%	14 130.01	13 522.92
LSTM	97.81%	977.18	4.84%	1856.21	1 371.02	90.47%	4 687.71	12.88%	6 163.92	5 110.33
SARIMA–LSTM	98.43%	820.38	3.94%	1 569.46	1 146.40	89.29%	4 126.54	11.92%	5 929.18	5 032.13
SARIMA–NARX	95.82%	1 239.12	6.11%	2 508.07	1805.93	85.96%	3 587.78	8.75%	4 519.34	3 935.07
SARIMA vs. LSTM	−1.31%	−12.37%	5.57%	21.47%	8.86%	−376.41%	65.64%	55.15%	56.38%	62.21%
SARIMA vs. SARIMA–LSTM	−1.96%	5.67%	23.25%	33.60%	23.79%	−370.24%	69.75%	58.50%	58.04%	62.79%
SARIMA vs. SARIMA–NARX	0.74%	−42.49%	−19.04%	−6.11%	−20.05%	−352.69%	73.70%	69.55%	68.02%	70.90%
LSTM vs. SARIMA–LSTM	−0.64%	16.05%	18.73%	15.45%	16.38%	1.29%	11.97%	7.47%	3.81%	1.53%
LSTM vs. SARIMA–NARX	2.03%	−26.81%	−26.06%	−35.12%	−31.72%	4.98%	23.46%	32.11%	26.68%	23.00%
SARIMA–LSTM vs. SARIMA–NARX	2.65%	−51.04%	−55.10%	−59.80%	−57.53%	3.73%	13.06%	26.63%	23.78%	21.80%

### The best-performing LSTM model and SARIMA-LSTM model

**Figure 4.**
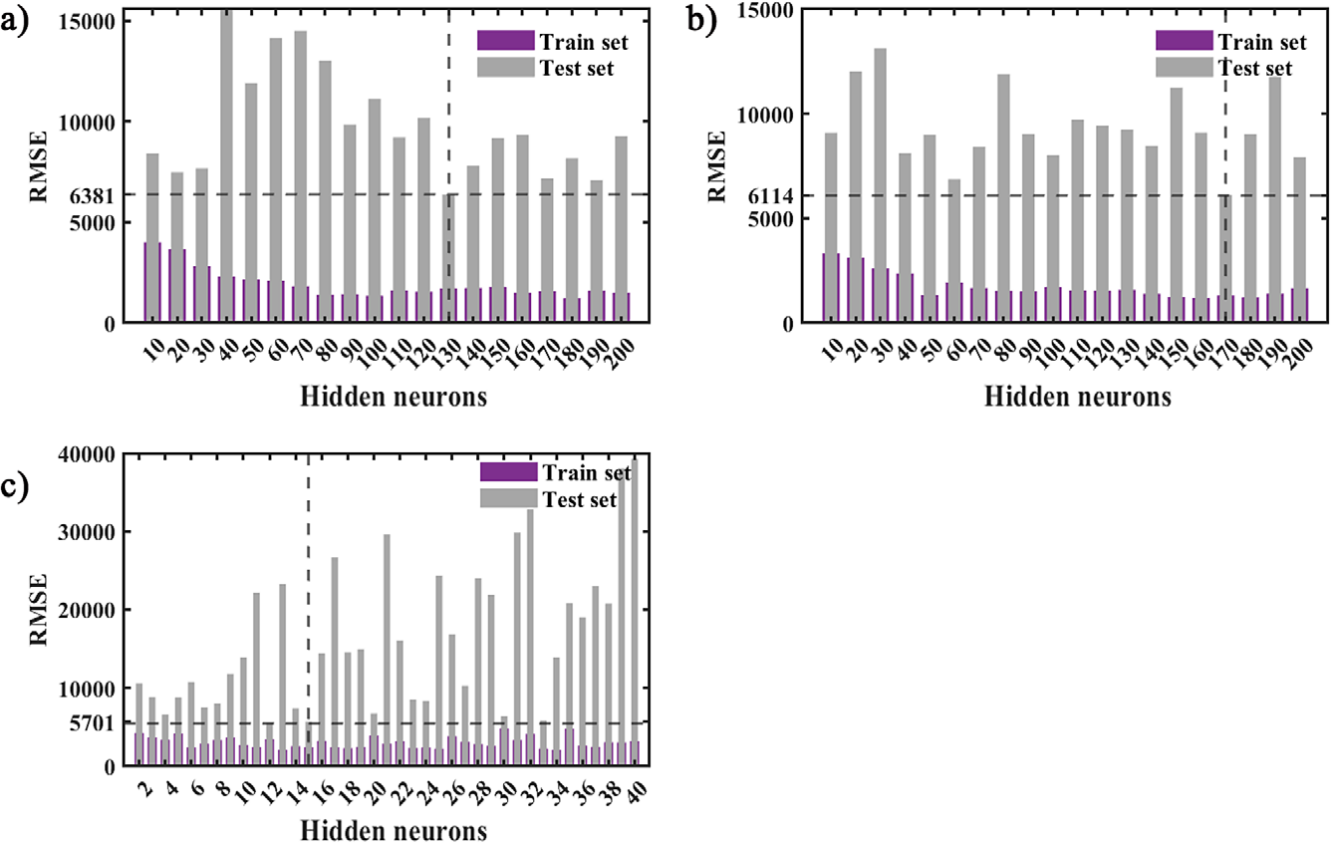
Fitting and predicting the performance of LSTM (a), SARIMA-LSTM (b), and SARIMA-NARX (c) models with different structures. *Note:* The dark blue and grey bars represent the RMSE values of the training and test sets, respectively

Upon establishing a constant number of maximum iterations and an initial learning rate, we systematically trained a series of LSTM neural network architectures differentiated by the quantity of hidden neurons embedded within them. These architectures were then employed to execute simulations on both the train and test datasets. Empirical evidence suggests that the architecture containing precisely 130 hidden neurons yielded superior predictive capabilities, as quantified by the RMSE on the test set, which registered a value of 6 381 (Figure 4a). Leveraging the LSTM model that demonstrated optimal performance characteristics, we engaged in an iterative process of retraining and forecasting. The conclusive model’s fitness was quantitatively assessed, with the results systematically tabulated in Table 1, and the corresponding simulation outputs alongside the residual diagnostics are illustrated in Figure 6. The residuals of the single SARIMA model are modelled quadratically using the LSTM approach, and then the output of the LSTM is summed with the fitted values of the SARIMA model to obtain the output of the hybrid SARIMA-LSTM model. Similar to the process of determining the structure of the LSTM model, we conducted training on LSTM models with varying numbers of hidden neurons, while maintaining fixed iterations and initial learning rates. Subsequently, all models were used to simulate the training and test sets. The results indicate that the model exhibits the best predictive performance when the number of hidden neurons is 170 (the RMSE value on the test set is 6 114, as shown in Figure 4b). The optimal SARIMA-LSTM model obtained was subjected to multiple rounds of training and prediction. The final goodness of fit results for the model are presented in Table 1, while the simulation results and residuals are depicted in Figure 6.

**Figure 5.**
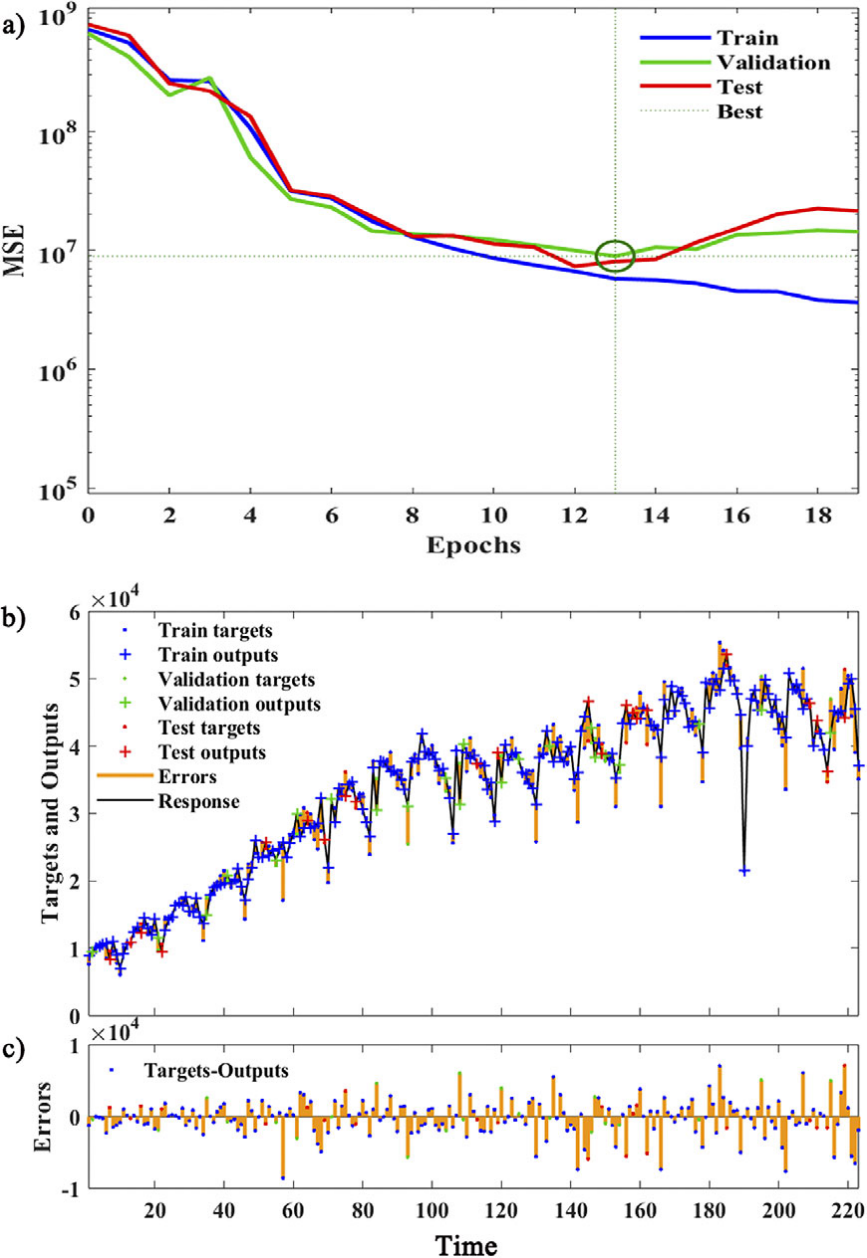
The simulation process for the training set by the SARIMA-NARX model. *Note:* (a) The variation of MSE for the training, validation, and test sets during the iteration process. (b) The error between the output values of each component data and the target values, while (c) provides a detailed display of the error magnitude. The blue, yellow, and red dots indicate the target values of the training set, validation set, and test set after simulation using the SARIMA-NARX model, and the blue, yellow, and red crosses denote the outputs of the training set, validation set, and test set, and the yellow stem denotes the error of fitting

**Figure 6.**
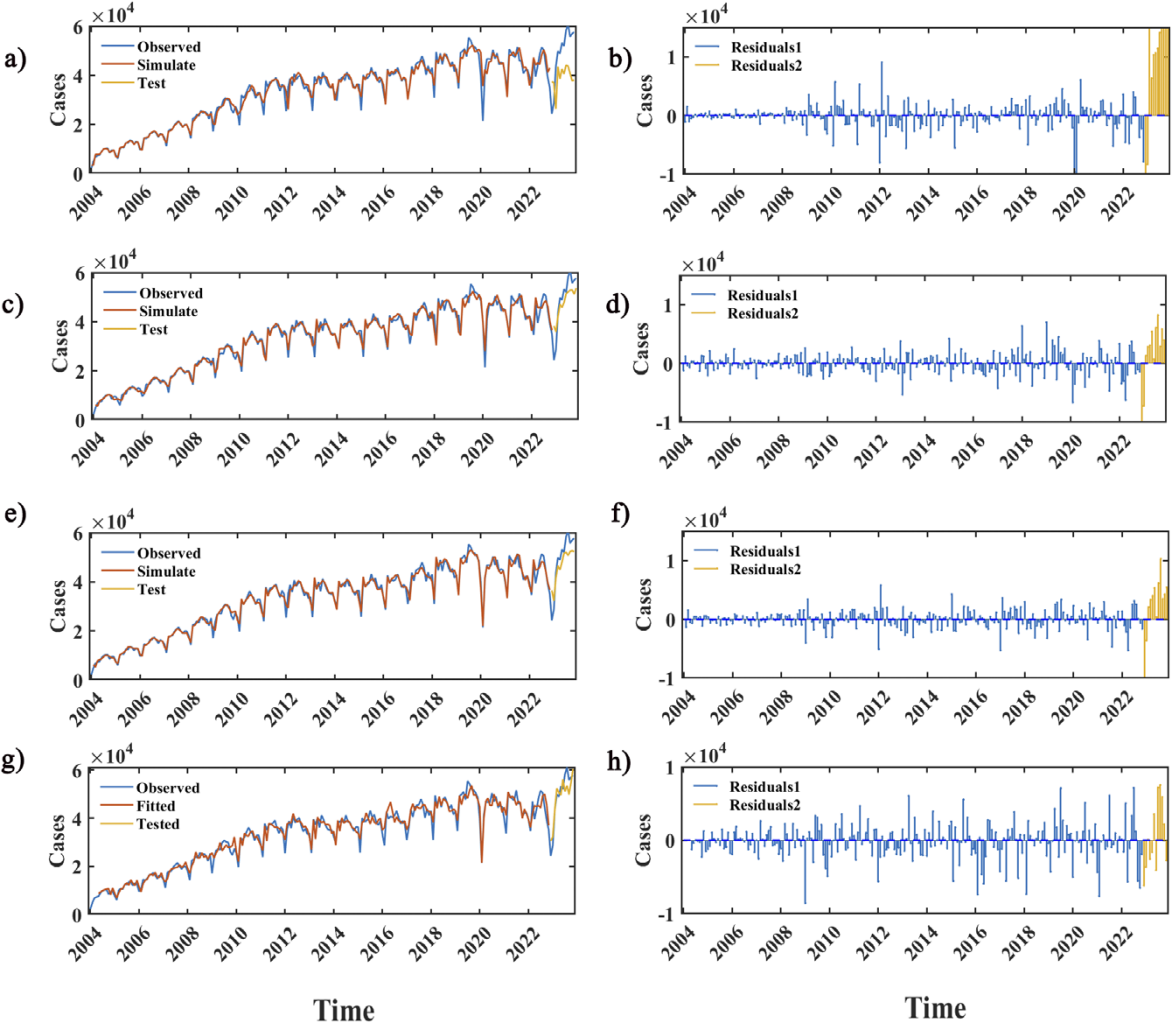
Fitting and forecasting performance of the SARIMA, LSTM, SARIMA-LSTM, and SARIMA-NARX models. *Note:* Panels (a), (c), (e), and (g) denote the fitting and predicting results using the SARIMA, LSTM, SARIMA-LSTM, and SARIMA-NARX models, respectively, the red and yellow curves represent the simulation values for the train set and test set of the TS. Panels (b), (d), (f), and (h) denote the residuals of the SARIMA, LSTM, SARIMA-LSTM, and SARIMA-NARX models, respectively, the blue and yellow stems represent the residuals for the train sets and test sets, respectively

### The best-performing SARIMA-NARX model

After several trials, we found that the best prediction performance was achieved when the number of hidden neurons was 30, with the minimal RMSE value(5701) of the test set (Figure 4c). So the structure of the SARIMA-NARX model was determined. Then an open-loop network was built. Before training, we divided the training set data into three parts, 80% for training, 10% for validation, and 10% for testing. At the 13th iteration of the model, the MSE value of the validation set reached its minimum and began to rise (Figure 5a). The output of the model training process and the errors are shown in Figure 5. After the training was completed, we converted the network into a closed-loop to perform the prediction for the forward 12 steps, and the goodness-of-fit was calculated for the training set and test set respectively (Table 1).

### Comparison of the fitting and predicting power of SARIMA, LSTM, SARIMA-LSTM, and SARIMA-NARX models

In conclusion, all four models exhibit high goodness-of-fit in the training set, with *R* ^2^ values exceeding 95%. Considering the comprehensive fit indices, the ranking of the goodness-of-fit from best to worst is SARIMA-LSTM model, LSTM model, SARIMA model, and SARIMA-NARX model. In terms of predictive performance, the SARIMA model performs the worst, almost unable to accurately predict future epidemic trends. The SARIMA-NARX model outperforms the other models, despite its *R* ^2^ value being slightly lower than the SARIMA-LSTM model. Its MAD value decreases by 352.69%, 4.98%, and 3.73% compared to SARIMA, LSTM, and SARIMA-LSTM models, respectively. Its MAPE value decreases by 73.7%, 23.46%, and 13.06% compared to SARIMA, LSTM, and SARIMA-LSTM models, respectively. The RMSE value decreases by 68.02%, 26.68%, and 23.78% compared to SARIMA, LSTM, and SARIMA-LSTM models, respectively. The MAE decreases by 70.90%, 23.00%, and 21.80% compared to SARIMA, LSTM, and SARIMA-LSTM models, respectively. Compared to the LSTM model, the SARIMA-LSTM model’s MAD, MAPE, RMSE, and MAE values decrease by 11.97%, 7.47%, 3.81%, and 1.53%, respectively. From the fitting curves of the four models, it can be observed that the LSTM, SARIMA-LSTM, and SARIMA-NARX models can all accurately predict future disease trends. Among them, the predictions from the LSTM and SARIMA-LSTM models are more similar. The predictive errors of the three models reach their maximum values in July and August 2023, and the predicted values are consistently lower than the actual data (Figure 6).

### Predictions of SARIMA-LSTM and SARIMA-NARX models

**Figure 7.**
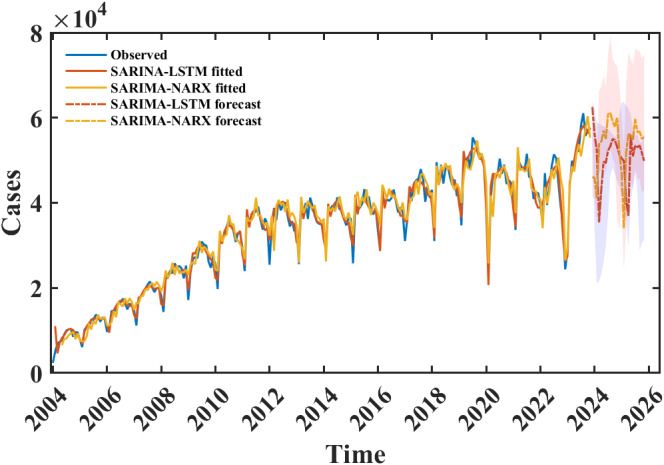
Prediction results from December 2023 to November 2025 of SARIMA-LSTM, and SARIMA-NARX models. *Note:* The light gray and blue areas respectively represent the forecast intervals of SARIMA-NARX and SARIMA-LSTM. The blue, red, and yellow curves represent the original data, the fitted values of the SARIMA-LSTM and SARIMA-NARX models, and the predicted values of the two models are represented by the red and yellow dashed lines

We remodelled the SARIMA, SARIMA-LSTM, and SARIMA-NARX models with all original data before predicting future time steps to ensure the accuracy of the predictions. The new SARIMA model was built first as a basis for the other two models, and after the sample size of the TS used for modelling was increased, the best-fitting SARIMA model was established as SARIMA(3,1,1) (4,1,0)_12_ with AIC and BIC values of 4 540.9 and 4 569.3, respectively. The construction of the hybrid SARIMA-LSTM and SARIMA-NARX models was then carried out based on the fitted values of the SARIMA model, and 20 times predictions of the monthly incidence of syphilis for the next 24 months (from December 2023 to November 2025) were made using the two models. In the forecasting application of the SARIMA-LSTM and SARIMA-NARX models, the architecture was preserved with an identical neuron configuration as employed during the training phase. Furthermore, an ensemble approach was implemented, where each model executed 20 times of forecasts. The extrema, specifically the maximal and minimal predictive values at each temporal increment, were systematically documented. This procedure was designed to facilitate the construction of predictive interval plots, enhancing the visualization and interpretation of forecast uncertainty. The results showed that the trends predicted by the SARIMA-LSTM and SARIMA-NARX models were similar, the forecasted values of the SARIMA-NARX model are slightly higher overall than those of the SARIMA-LSTM. The peak number of monthly incidences appeared in July and August of 2024 during the prediction period (Figure 7).

## Discussion

Syphilis is a highly insidious STD and has long-lasting damage to the human body. As the STD with the highest incidence in China and has been increasing for more than 10 years, it is essential to fit and predict the incidence data of syphilis. This will help the government to formulate relevant public health policies in advance, and rationalize the allocation of public health resources to avoid widespread infection in the population. After seasonal and long-term trend decomposition of the sample data, we found that the syphilis prevalence in China has been growing rapidly from 2004 to 2012, since then onset growth has slowed until the end of 2019. The incidence is generally higher in the summer and fall of each year, and after the outbreak of COVID-19, the growth trend slowed down but the overall incidence level is still high. Undoubtedly, the stringent public health and social interventions adopted in China’s response to the COVID-19 process have played a specific role in curbing the transmission of syphilis, but as China gave up the *zero-COVID* strategy for the normalization of prevention and control of COVID-19, the epidemiology trend of syphilis need to be reassessed. In February 2020, the number of monthly reported cases of syphilis was 21 448, a decrease of 53.1% compared with January 2020, making it the month with the lowest number of reported cases in the past 10 years, probably since at this time, lockdowns and restrictions on gatherings and travel were imposed in the nationwide and most of the inhabitants and healthcare workers were amid their Lunar New Year vacations, which impacted the detection of the disease and the reporting of cases [28]. Then, China implemented the *zero-COVID* strategy, and population mobility was significantly reduced, resulting in a blockage of interpersonal transmission of the disease, which has resulted in a smooth fluctuation in the number of reported cases, rather than continuing to grow.

The seasonality of syphilis epidemic behaviour can be related to sexual behaviours in Chinese populations and the patients’ clinical attendance [29]. Some studies have found that in early spring, shortly after the end of the Chinese New Year, there is often a mass migration of Chinese populations, many of whom are returning from rural to urban jobs, and that this group behaviour is often accompanied by an increase in sexual behaviour [30, 31]. Influenced by traditional Chinese beliefs, the willingness to diagnose and seek medical care around the Spring Festival is lower than at other times of the year, but in the summer months, graduated students and job seekers undergo mandatory medical check-ups before enrolling in school and the military as well as entering the workforce, which included serological testing for the virus [30], in addition, changes in hormone levels may lead to an increase in sexual behaviour during specific seasons [31], leading to a peak in incidence in the summer and fall, which may explain the seasonal pattern of syphilis in China.

As a traditional mathematical model, SARIMA model is widely used in the analysis of TS data [32–34], but one of the applicable conditions is that the research data must be smooth, so it is often necessary to transform the data to achieve the modelling conditions, this process will often lose part of the information contained in the original data, for the fitting and prediction of complex nonlinear data, SARIMA model may not be able to precisely. The emergence of neural network algorithms has improved this deficiency, as the neural network model involves a large number of neurons in computation, and the overall system output is calculated through the interactions between neurons. As a result, the network possesses good robustness, such that even if there are errors in a certain part of the network, it will only reduce the adaptability of the network rather than causing significant errors.

Through iterations, the connections between neurons can be adjusted, allowing specific logical operations or nonlinear computations to be performed from complex or imprecise data. Although the LSTM and NARX models have unique advantages in TS data modelling, a single neural network model still has limitations in its usage. LSTM and NARX neural networks simply use known inputs to estimate the current output, which may affect the accuracy of their predictions and inferences [10], especially when time variables are crucial, particularly in cases where TS exhibit seasonality. Therefore, we attempt to establish SARIMA-LSTM and SARIMA-NARX combined models to explain the relationship between the fitted values of the SARIMA model and the sample data, emphasizing the time variable. As the SARIMA model has a good capturing ability for periodic fluctuations, while LSTM and NARX excel in capturing nonlinear oscillations. Our research results also demonstrate that the combined model has better predictive capabilities than a single model, indicating that the combined model can integrate the strengths of individual single models.

In terms of predictive power, predictive models are considered perfect when the MAPE value is less than 5% [35]. Models with MAPE values in the range of 5%–10% are considered high-precision models; models with MAPE values in the range of 10%–20% are considered good models [36]. Although the thoughts of building the hybrid SARIMA-LSTM and SARIMA-NARX models in this study are different, they are both a quantitative description of the relationship between the output of SARIMA and the actual onset data using the LSTM and NARX models, which can be regarded as the SARIMA model nested in the essentially neural network models and thus the results are comparable. However, in determining the best-fitting SARIMA-LSTM and SARIMA-NARX models, we found that the prediction performance of SARIMA-NARX models is not as stable as that of SARIMA-LSTM models when different parameters are used for the model construction, and therefore, there is a higher demand for parameter selection, otherwise, the prediction error will be large. For the actual data in July and August 2023, both the SARIMA-LSTM and SARIMA-NARX models exhibit underestimation, which may be attributed to the fact that the data for these 2 months exceeds all the data within our study period and can be considered as outliers. The predictions of the SARIMA-LSTM and SARIMA-NARX showed that the results of the two models had similar trends, suggesting that syphilis epidemiological trends in China will remain characterized by a high and stable level of epidemiological trends in the future. From May 2023 to November 2023, the reported number of cases for each month was significantly higher than the number of cases during the corresponding period, indicating the need for early intervention measures to prevent potential risks. Since the social and economic impacts of COVID-19 have not yet been eliminated, this study can provide a cost-effective tool for China and worldwide, which can help to identify trends in disease prevalence, rationalize the allocation of public health resources, avoid the waste of medical resources, and protect people’s health.

## Limitations

Admittedly, this study has several limitations. First, although the sample data were acquired from the official health administration in China, they were reported and aggregated by regional healthcare institutions at all levels, and between December 2019 and December 2022, the Chinese government has taken strict public health measures in response to the COVID-19 pandemic, which could lead to a decrease in the accessibility of people at high risk of syphilis infection to seek medical inspection, so the data may be subject to reporting bias. Second, although the LSTM model has high fitting accuracy, the training progress and parameter optimization of the model require a lot of time because of the complex structure of the LSTM model. Third, the TS model can only be used for short-term prediction, and the accuracy will be reduced if a long-term prediction is performed, so the data needs to be updated frequently to optimize the model. Finally, the determination of model parameters for LSTM and NARX models currently lacks a well-established theoretical framework and often relies on heuristic and empirical methods. While model selection is typically based on the evaluation of prediction performance using a test set, this approach may not adequately assess the model’s generalization ability to unforeseen data. Therefore, caution is advised when extrapolating the predictions of future disease incidence from this study.

## Conclusions

The hybrid SARIMA-NARX and SARIMA-LSTM methods predict syphilis cases more accurately than the basic SARIMA and LSTM methods, so that can be used for governments to rationally allocate health resources and develop long-term syphilis prevention and control programs. In addition, the predicted cases still maintain a fairly high level of incidence, so there is an urgent need to develop more comprehensive prevention and control, and intervention strategies.

## Supporting information

Wang et al. supplementary material Wang et al. supplementary material

## Data Availability

The sample data are available at http://www.nhc.gov.cn/jkj/new_index.shtml and https://www.cnki.net/.

## Supplementary material

For supplementary material accompanying this paper visit http://dx.doi.org/10.1017/S0950268824000694. click here to view supplementary material
